# Development and characterization of 16 polymorphic microsatellite markers from Taiwan cow-tail fir, *Keteleeria davidiana* var. *formosana* (Pinaceae) and cross-species amplification in other *Keteleeria* taxa

**DOI:** 10.1186/1756-0500-7-255

**Published:** 2014-04-23

**Authors:** Chin-Shang Ho, Huei-Chuan Shih, Ho-Yih Liu, Shau-Ting Chiu, Mei-Hui Chen, Li-Ping Ju, Ya-Zhu Ko, Yu-Shen Shih, Chaur-Tzuhn Chen, Tsai-Wen Hsu, Yu-Chung Chiang

**Affiliations:** 1 Graduate Institute of Bioresources, National Pingtung University of Science and Technology, Pingtung 91201, Taiwan; 2 Department of Nursing, Meiho University, Pingtung 912, Taiwan; 3 Department of Biological Sciences, National Sun Yat-sen University, Kaohsiung 804, Taiwan; 4 Department of Biology, National Museum of Natural Science, Taichung 404, Taiwan; 5 Conservation Division, Forestry Bureau, Council of Agriculture, Executive Yuan, Taipei 100, Taiwan; 6 Botanical Garden Division, Taiwan Forestry Research Institute, Taipei 100, Taiwan; 7 Department of Life Sciences, National Chung Hsing University, Taichung 40227, Taiwan; 8 Department of Forestry, National Pingtung University of Science and Technology, Pingtung 912, Taiwan; 9 Endemic Species Research Institute, Nantou 552, Taiwan

**Keywords:** *Keteleeria*, Conservation, Microsatellites, Simple sequence repeat markers, Pinaceae

## Abstract

**Background:**

*Keteleeria davidiana* var. *formosana* (Pinaceae), Taiwan cow-tail fir, is an endangered species listed on the IUCN Red List of Threatened Species and only two populations remain, both on the Taiwan Island. Sixteen polymorphic microsatellite loci were developed in an endangered and endemic gymnosperm species, *Keteleeria davidiana* var. *formosana*, and were tested in an additional 6 taxa, *K. davidiana* var. *calcarea*, *K. davidiana* var. *chienpeii*, *K. evelyniana*, *K. fortunei*, *K. fortunei* var. *cyclolepis*, and *K. pubescens*, to evaluate the genetic variation available for conservation management and to reconstruct the phylogeographic patterns of this ancient lineage.

**Findings:**

Polymorphic primer sets were developed from *K. davidiana* var. *formosana* using the modified AFLP and magnetic bead enrichment method. The number of alleles ranged from 3 to 16, with the observed heterozygosity ranging from 0.28 to 1.00. All of the loci were found to be interspecifically amplifiable.

**Conclusions:**

These polymorphic and transferable loci will be potentially useful for future studies that will focus on identifying distinct evolutionary units within species and establishing the phylogeographic patterns and the process of speciation among closely related species.

## Findings

### Background

For conservation management, the evaluation of genetic diversity and the identification of distinct evolutionary units in endangered taxa should allow for the development of more efficient conservation strategies [1-3]. Over the past century, human activities associated with the lumber industry and agriculture have destroyed and fragmented forest habitats at altitudes of 500–2500 m on Taiwan Island. Several gymnosperm species have decreased in population size and are now under threat, including *Cycas taitungensis*[4-6], *Podocarpus nakaii*[1], *Amentotaxus formosana*[2], and *Keteleeria davidiana* var. *formosana*[7]. Extreme population declines can affect the genetic diversity of a species [8]. The loss of genetic diversity carries serious evolutionary concerns regarding both the ability to adapt to changes on oceanic islands and longer-term interactions with other organisms [9]. Thus, molecular markers are used to evaluate endangered species in greater depth, with the purpose of applying this genetic diversity information to the conservation and restoration of biodiversity [10].

*Keteleeria davidiana* var. *formosana* (Pinaceae), Taiwan cow-tail fir, is an endangered species listed on the IUCN Red List of Threatened Species [11] and derived from *K. davidiana*, including var. *davidiana* and var. *calcarea*, occurred in China by geographical disjunction and leaf scars obscurely or obviously protruding on branchlets [12]. The taxa has only two wild populations, the Dawu Taiwan *Keteleeria* Forest Reserve (Dawu) and the Pinglin Taiwan *Keteleeria* Nature Reserve (Pinglin), which are located in Northern and Southern Taiwan, respectively [13]. A previous inventory study has found that less than a hundred individuals remain in the wild, few seedlings are found in the field, and adult trees are vulnerable to lightning damage or death [14]. Additionally, continuous logging over the past century in the low-elevation broadleaf forests has restricted *K. davidiana* var. *formosana* to narrow habitats and has decreased its population size [15]. Therefore, the conservation strategies for *Keteleeria davidiana* var. *formosana* should include *in situ* protection and management in conjunction with *ex situ* approaches such as clonal orchards. Hence, the population genetics of this species should be evaluated before *in situ* and *ex situ* conservation management begins. In this study, we developed polymorphic microsatellite loci from Taiwan cow-tail fir to estimate the population structure within the species and to identify distinct evolutionary units within the population. In addition, the transferability of the microsatellite primers developed in this study was tested in other taxa, specifically, three species and three varieties of the *Keteleeria* genus.

## Methods

### Sampling and DNA extractions

Twenty-five individuals were sampled from each of the two remaining populations of *K. davidiana* var. *formosana* from Dawu and Pinglin, Taiwan (Table 1). To test the transferability of the markers, two to three individuals of three species, *K. pubescen*s, *K. fortune*, and *K. evelyniana*, and three varieties, *K. fortunei* var. *cyclolepis*, var. *calcarea*, and *K. davidiana* var. *chienpeii*, were sampled from the field. Permission for tissue collection was obtained from the Council of Agriculture, Republic of China, authorities. All taxa of the *Keteleeria* genus are diploid with 24 chromosomes [13]. The sample size, location, and storage herbarium for the voucher specimens are listed in Table 1. Total genomic DNA was extracted from silica-dried leaf powder using the Plant Genomic DNA Extraction Kit (RBC Bioscience, Taipei, Taiwan).

**Table 1:** **Sample location for each species, variety, and population of the *Keteleeria *** **species**

**Species**	**Location**	**Sample size**	**Latitude**	**Longitude**	**Herbarium**
*K. davidiana* var. *formosana*	Pinglin, Taiwan	25	N 24°53′28″	E 121°42′12″	TAIF
	Dawu, Taiwan	25	N 22°21′23″	E 120°47′27″	TAIF
*K. fortunei* var. *cyclolepis*	Fujian, China	3	N 27°09′18″	E 118°13′03″	TAIF
*K. pubescens*	Guizhou, China	3	N 26°30′54″	E 108°03′52″	TAIE
*K. davidiana* var. *calcarea*	Guangxi, China	3	N 25°12′47″	E 110°11′50″	TAIE
*K. davidiana* var. *chienpeii*	Guizhou, China	3	N 26°20′26″	E 106°41″52″	TAIE
*K. fortunei*	Fujian, China	3	N 26°09′57″	E 119°02′19″	TAIF
*K. evelyniana*	Yunnan, China	3	N 23°42′55″	E 105°07′24″	TAIE

Sample size, location, coordinates, and voucher specimens are indicated.

*Note*: TAIF = the herbarium of the Taiwan Forestry Research Institute; TAIE = the herbarium of the Taiwan Endemic Species Research Institute.

### Isolation of microsatellite DNA loci and identification

To isolate the microsatellites efficiently, we used the modified AFLP [16] and magnetic bead enrichment method [1,17,18] to select microsatellite loci. Genomic DNA from one individual of *K. davidiana* var. *formosana* was treated with the restriction enzyme *Mse*I (Promega, Madison, Wisconsin, USA) and separated using 1% Nusieve® 3:1 agarose gel (FMC Bio Products, Rockland, ME, USA) electrophoresis. The cut DNA fragments ranged from 400 to 1000 bps, and the purified fragments were extracted from the agarose gel using the HiYield™ Gel PCR DNA Fragments Extraction Kit (RBC Bioscience). Purified DNA fragments were ligated to a double-stranded *Mse*I-adaptor (complementary oligo A: 5′-TACTCAGGACTCAT-3′; 5′phosphorylated oligo B: 5′-GACGATGAGTCCTGAG-3′) using the Quick Ligation™ Kit (New England Biolabs, MA, USA) at 25°C for 5 minutes. The ligation product was used as the template DNA for the enrichment of the partial genomic library, and the adaptor-specific primers, named Mse I-N (5′-GATGAGTCCTGAGTAAN-3′), were used to perform 20 cycles of prehybridization PCR amplification. The PCR mixture contained 20 ng template DNA, 10 pmol adapter-specific primer, 2 μL 10 × reaction buffer, 2 mM dNTP mix, 2 mM MgCl_2_, 0.5 U *Taq* DNA polymerase (Promega), and sterile water was added to reach a total volume of 20 μL. The PCR protocol was set at 94°C for 5 min, followed by 18 cycles of 94°C for 30 s, 53°C for 1 min, and 72°C for 1 min, using a Labnet MultiGene 96-well Gradient Thermal Cycler (Labnet, Edison, New Jersey, USA). The amplicons were denatured and hybridized to four separated 5′-biotinylated oligonucleotide probes, (AG)_15_, (AC)_15_, (TCC)_10_, and (TTG)_10_, in 250 μL of hybridization solution at 68°C for 1 h, and 1 mg of Streptavidin MagneSphere Paramagnetic Particles (Promega) was added to the mixture solution to capture the hybridizations at 42°C for 2 h. The four enriched microsatellite DNA fragments were washed with high-salt and low-salt solutions and then used as templates for 25 cycles of PCR amplification using the adaptor-specific primers; the amplification protocol was same as that used for the prehybridization PCR. The PCR products were purified using the HiYield™ Gel PCR DNA Fragments Extraction Kit (RBC Bioscience) and then cloned using the pGEM®-T Easy Vector System (Promega). The plasmid DNAs that were purified from white colonies were digested by the restriction enzyme *Eco*RI (Promega) and screened using 1% agarose electrophoresis to determine the size of the inserted DNA fragments. In total, 670 white colonies were selected to purify plasmids for sequencing from *Keteleeria davidiana* var. *formosana.* The sequencing cocktail contained 200 ng plasmid DNA as the template DNA, 0.5 mM T7 or SP6 primers, 4 μL Big Dye Terminator v3.1 Ready Reaction Premix Reactions (Applied Biosystems, Carlsbad, California, USA), and sequencing buffer in a total volume of 20 μL. Selected plasmid DNAs were sequenced in both directions using an ABI PRISM® 3700 DNA Sequencer (Applied Biosystems). These sequences, including their microsatellite loci, were recognized using Tandem Repeats Finder version 4.07b [19], and a pair of specific primers for each microsatellite locus was designed using FastPCR software version 6.4.18 [20].

### DNA amplification and genotyping

The optimal annealing temperature was evaluated by gradient PCR using a Labnet MultiGene 96-well Gradient Thermal Cycler (Labnet) for a temperature range from 50°C to 65°C. For each *Keteleeria* taxon, two to three individuals were evaluated for the optimal annealing temperature. The polymorphism of the two remaining populations of *K. davidiana* var. *formosana* was evaluated using 25 individuals from each population (Table 1). To test the optimal annealing temperature, a 20 μL reaction mixture containing 20 ng template DNA, 0.2 μM each of the reverse and forward primers, 2 μL 10 × reaction buffer, 2 mM dNTP mix, 2 mM MgCl_2_, 0.5 U Taq DNA polymerase (Promega), and sterile water was amplified using a Labnet MultiGene 96-well Gradient Thermal Cycler (Labnet). The PCR program was set at 94°C for 5 min, followed by 30 cycles of 94°C for 40 s, a temperature gradient ranging from 50 to 65°C for 60 s, and 72°C for 60 s, and a final extension of 72°C for 10 minutes [21]. Amplicons were checked by 1% agarose electrophoresis to isolate the target DNA bands, which were confirmed by sequencing [18]. To examine the genetic polymorphisms, the PCR cocktail was amplified using the previously described program with the temperature gradient replaced by the optimal annealing temperature (*Ta*). The amplicons were checked by 1% agarose electrophoresis to isolate the target DNA bands, which were confirmed by sequencing [18]. The amplicons were separated by electrophoresis on a 10% polyacrylamide gel (acrylamide: bisacrylamide 29: 1, 80 V for 14–16 hours) using a 25 or 50 bp DNA Step Ladder (Promega) to determine the allele size. The bands were then imaged under UV light using the Flo Gel FGIS-3 fluorescent gel image system (Top BIO Co., Taipei, Taiwan), and the sizes of the PCR products were identified using Quantity One software version 4.62 (Bio-Rad Laboratories, Hercules, California, USA).

### Data analysis

The genetic variation indices, including the number of alleles (*Na*), the number of effective alleles (*Ne*), the observed heterozygosity (*Ho*) and expected heterozygosity (*He*), Shannon’s information index (*H*), and fixation index (*F*_*IS*_) were estimated using GenAlEx version 6.5 [22]. The Hardy–Weinberg equilibrium (*H*_*WE*_) was tested using Arlequin software version 3.5.1.2 [23].

## Results and discussion

### Development of polymorphic microsatellite markers

A total of 392 microsatellite loci containing repeat motifs were discovered, with maximum and minimum lengths of 992 bps and 119 bps, respectively, and an average sequence length of 477 bps. We designed 102 primer pairs between the up- and down-flanking regions of the motifs based on the primer design parameters computed using FastPCR software version 6.4.18 [20]. To test the optimal annealing temperatures, which were obtained using gradient temperature PCRs, template DNA was derived from three individuals of each of the *Keteleeria* taxa, which are listed in Table 1. We tested the polymorphisms for *K. davidiana* var. *formosana* using 25 individuals from each population. Finally, we selected 16 polymorphic loci from the 102 microsatellites based on the detection of unambiguous polymorphic amplicons using fixed annealing temperature PCR and a polyacrylamide gel genotyping protocol. The characteristics of the 16 polymorphic microsatellite loci are listed in Table 2. Of the 16 loci, 11 are complete microsatellite loci, consisting of 5 with a dinucleotide motif, 5 with a trinucleotide motif, and 1 with a tetranucleotide motif. Of the 5 remaining loci, 3 carried a compound motif and 2 carried an interrupted motif. The sequences of 16 loci reported in this paper are available from GenBank (accession numbers: HG518488–HG518503) (Table 2).

**Table 2:** **Summary of general information for the 16 polymorphic microsatellite loci isolated from *Keteleeria davidiana *** **var. *formosana *** **and their transferability to six *Keteleeria *** **taxa**

**Locus**	**Repeat motif**	**Primer sequence(5′-3′)**	**Allele size (bps)**	***Ta*** **(°C)**							**Genbank accession no.**	**Cross amplification**
				**TW**	**cy**	**pu**	**ca**	**cp**	**fo**	**ev**		
TW-5	(TG)_15_	F: TTGTCTAGGTATTTGTGCCC	116	54	54	54	54	54	54	54	HG518488	7/7
		R: AAACGCAGTATAAGACAGGC										
TW-49	(CTCC)_9_	F: TCACTCCCTATATCCCTAGC	99	50	50	50	50	50	50	50	HG518489	7/7
		R: GGAGAGAGAGATTGTGTTTG										
TW-69	(CT)_27_(TATTTGA)_3_	F: CCTATATCCTCCCCCACCT	260	54	54	54	54	54	54	54	HG518490	7/7
		R: AGGTATAGAGGGGGATAGTG										
TW-107	(CA)_4_(AG)_14_	F: AACCAAGGATGAAACCCTAG	153	54	54	54	54	54	54	54	HG518491	7/7
		R: AAGTGAAAGACATAGTGAGG										
TW-172	(GA)_30_	F: AGACAAAGGAGAATGGTGGG	165	50	50	50	50	50	50	50	HG518492	7/7
		R: CCATGCCATGGGTAAGATAG										
TW-195	(CT)_18_	F: ATCTTCATCACTCTCCCACC	170	54	54	54	54	54	54	54	HG518493	7/7
		R: AAGGGGAAAGTGAGAGAGGG										
KE-6	(AC)_15_	F: ACGCGAGGTTTCGGGTGGCA	192	54	54	54	54	54	54	54	HG518494	7/7
		R: CTGGGTACTGGTGCCCTGT										
KE-54	(AC)_15_	F: ACCTTGCAAACTCACCAGCT	271	52	52	52	52	52	52	52	HG518495	7/7
		R: CTATGGCTAGGTTTCCTGGGT										
TCC-6	(AAC)_9_	F: TGTATGGTGTGTCAGATGC	342	56	56	56	56	56	56	56	HG518496	7/7
		R: CCTCCTTGTGGAGTTGGCA										
TCC-31	(TTG)_5_(TTC)_6_	F: CTTCAAAGCTGCTCGACTC	285	55	55	55	55	55	55	55	HG518497	7/7
		R: GTTTGCTTGAGGAACAGGT										
TCC-55	(GAA)_6_(CAA)_12_	F: CTCTTGAAGAACAGGTTGAG	288	54	54	54	54	54	54	54	HG518498	7/7
		R: CTTCAAAGCTGCTCGGCTCT										
TCC-139	(GAA)_13_	F: CCTACAAGGTTGACCCCGT	220	54	54	54	54	54	54	54	HG518499	7/7
		R: TGCTCGCCAATGCATGACAAG										
TCC-245	(GAA)_13_	F: ACGTTTGAGGAGCAGGTTG	255	50	50	50	50	50	50	50	HG518500	7/7
		R: TGAGAGATGTTCCTGTTCTG										
TTG-11	(CAA)_10_	F: GTTTTGTAGCTCTGTTGGAC	251	50	50	50	50	50	50	50	HG518501	7/7
		R: CCATATGACCTAGCTTCCCA										
TTG-49	(AGG)_8_	F: GAGATGACAGAGGCTGCTG	224	55	55	55	55	55	55	55	HG518502	7/7
		R: GCTAGTGGGTGCACTAGGT										
TTG-54	(GA)_15_(GAA)_2_	F: CTCGTGCTAACCCTTGATC	222	50	50	50	50	50	50	50	HG518503	7/7
		R: GGCTACCTTAGGATGTGTGCA										

*Note:* TW = *K. davidiana* var. *formosana;* cy = *K. fortunei var.cyclolepis;* pu = *K. pubescens;* ca = *K. davidiana* var. *calcarea;* cp = *K. davidiana *var. *chien-peii;* fo = *K. fortune;* ev = *K. evelyniana;* F = the forward primer; R = the reverse primer; *Ta *= optimized annealing temperature.

### Genotyping and population genetics analysis

To examine the extent of genetic polymorphism at each locus, 25 individuals were collected in the field from each of the two remaining populations of *K. davidiana* var. *formosana* (Table 1). For the 16 new polymorphic microsatellite loci, the number of alleles per locus (*Na*) and the number of effective alleles (*Ne*) ranged from 5 to 14 and 2.59 to 11.25 in the Dawu population, respectively, and from 3 to 16 and 2.14 to 10.59 in the Pinglin population, respectively (Table 3). The observed and expected heterozygosity (*Ho* and *He*, respectively) varied from 0.44 to 1.00 (average of 0.68) and 0.61 to 0.91 (average of 0.82) in the Dawu population, respectively, and from 0.28 (average of 0.63) to 0.80 (average of 0.78) in the Pinglin population. The means of Shannon’s information index (*H*) and the fixation index (*F*_*IS*_) were 1.93 and 0.175 and 1.73 and 0.188 in the Dawu and Pinglin populations, respectively (Table 3). Significant deviations from Hardy–Weinberg equilibrium (*H*_*WE*_) were detected at 6 and 3 loci in the two populations, respectively, and these deviations were attributed to the heterozygote deficiency of the endangered species (Table 3). A total of 52 and 29 private alleles were observed in the Dawu and Pinglin populations, respectively, revealing population differentiation between the two remaining populations of the species.

**Table 3:** **Genetic diversity characteristics of the 16 polymorphic microsatellite loci tested on two populations of *Keteleeria davidiana *** **var. *formosana***

	***K. davidiana *** **var. *formosana***											
	**Dawu population (N = 25)**						**Pinglin population (N = 25)**					
**Locus**	***Na***	***Ne***	***Ho***	***He***	***H***	***F***_***IS***_	***Na***	***Ne***	***Ho***	***He***	***H***	***F***_***IS***_
TW-5	5	2.59	0.44	0.61	1.18	0.284	3	2.68	0.56	0.63	1.03	0.106
TW-49	7	3.93	0.68	0.75	1.55	0.088	3	2.14	0.48	0.53	0.90	0.100
TW-69	8	5.30	0.68	0.81	1.84	0.162	7	4.73	0.64	0.79	1.71	0.189
TW-107	7	4.36	0.56	0.77*	1.68	0.273	4	3.24	0.52	0.69	1.28	0.248
TW-172	6	4.63	0.68	0.78	1.63	0.133	3	2.35	0.52	0.57	0.97	0.093
TW-195	5	4.96	0.72	0.80	1.61	0.098	6	5.00	0.72	0.80	1.66	0.100
KE-6	12	9.69	0.60	0.90*	2.37	0.331	8	7.27	0.28	0.86*	2.03	0.675
KE-54	10	7.62	0.68	0.87	2.14	0.217	8	7.23	0.68	0.86	2.02	0.211
TCC-6	10	5.79	0.80	0.83	1.98	0.033	10	8.56	0.80	0.88	2.22	0.094
TCC-31	8	5.76	0.72	0.83	1.89	0.129	9	5.73	0.72	0.83	1.96	0.128
TCC-55	10	8.50	0.68	0.88	2.22	0.229	10	8.22	0.76	0.88	2.18	0.135
TCC-139	6	4.92	0.64	0.80*	1.67	0.197	10	5.73	0.56	0.83*	1.96	0.322
TCC-245	8	6.04	0.72	0.83	1.91	0.137	4	3.33	0.72	0.70	1.29	-0.029
TTG-11	13	7.76	0.76	0.87*	2.28	0.128	8	6.19	0.72	0.84	1.93	0.141
TTG-49	14	11.26	1.00	0.91*	2.51	-0.097	10	6.87	0.80	0.85	2.06	0.064
TTG-54	13	9.92	0.48	0.90*	2.42	0.466	16	10.59	0.52	0.91*	2.55	0.426
Mean	8.88	6.44	0.68	0.82	1.93	0.175	7.44	5.62	0.63	0.78	1.73	0.188

*Significant deviation from Hardy-Weinberg equilibrium: P < 0.05.

The number of different alleles (*Na*), number of effective alleles (*Ne*), observed heterozygosity (*H*_*O*_), expected heterozygosity (*He*), Shannon’s information index (*H*), and fixation index (*F*_*IS*_) are reported.

The genetic variability, including the means of the observed and expected heterozygosity (Table 3), of *K. davidiana var. formosana* was high compared with that of other Pinaceae species such as *Pinus koraiensis* (0.38 and 0.57) [24] and *P. massoniana* (0.27 and 0.65) [25,26], but was similar to that of *P. pinaster* (0.65 and 0.83) [27]. Unfortunately, no data for other *Keteleeria* taxa are available for the comparison of genetic variability. However, the high observed and expected heterozygosity values implied that the species was historically larger in population size and was more common in low-elevation forests than it is at present [7].

### Test of transferability

To test the transferability of these microsatellite loci, we tested these primers in six other cow-tail fir taxa: *K. fortunei* var. *cyclolepis*, *K. pubescens*, *K. davidiana* var. *calcarea*, *K. davidiana* var. *chienpeii*, *K. fortune*, and *K. evelyniana* (Table 1). Two to three samples of each taxon were used in the evaluation of cross-amplification. All loci were transferable to the six taxa of *Keteleeria* (Table 4), and the annealing temperatures are listed on Table 2. However, few alleles were recovered in the six other *Keteleeria* taxa sampled here were caused by a small sampling effect. Nonetheless, the systematic and phylogeographic patterns among the *Keteleeria* species remain unclear. The transferability of these microsatellite loci among different *Keteleeria* taxa indicates the usefulness of these molecular genetic markers for interspecific and intraspecific research on such topics as phylogeography, speciation and introgression.

**Table 4:** **Result of cross-species transferability in six *Keteleeria *** **taxa using the 16 microsatellite primers developed from *Keteleeria davidiana *** **var. *formosana***

**Locus**	***K. fortunei *** **var. *cyclolepis *** **(N = 3)**	***K. pubescens *** **(N = 3)**	***K. davidiana *** **var. *calcarea *** **(N = 3)**	***K. davidiana *** **var. *chienpeii *** **(N = 2)**	***K. fortunei *** **(N = 3)**	***K. evelyniana *** **(N = 3)**
TW-5	1	2	1	2	1	1
TW-49	2	2	2	1	1	2
TW-69	3	2	2	2	1	2
TW-107	3	1	2	2	1	1
TW-172	2	1	2	1	1	2
TW-195	2	2	3	2	2	1
KE-6	2	3	3	2	2	2
KE-54	3	3	2	1	2	2
TCC-6	3	3	3	2	3	3
TCC-31	2	3	2	2	2	1
TCC-55	3	2	1	2	1	2
TCC-139	2	3	2	1	2	2
TCC-245	2	2	2	2	2	1
TTG-11	3	3	3	2	3	3
TTG-49	3	2	3	2	3	3
TTG-54	2	2	3	2	2	2

For loci that were successfully amplified, the number of alleles is given.

## Conclusions

For conservation purposes, 16 new polymorphic microsatellite loci were developed from *K. davidiana var. formosana*. The genetic variation indices evaluated using these 16 polymorphic microsatellite loci for the two remaining populations of this endangered species are potentially useful for future studies that will focus on identifying distinct evolutionary units within the populations for conservation management. The interspecies transferability of these microsatellite loci may also be useful for future research aiming to reconstruct the phylogeographic patterns and the process of speciation among closely related species.

## Availability of supporting data

The microsatellite sequences are available through the National Centre for Biotechnology Information (see http://www.ncbi.nlm.nih.gov/). The accession numbers on the repository are the following: GenBank accession number HG518488 through HG518503.

## Competing interests

The authors declare that they have no competing interests.

## Authors’ contributions

T-WH and Y-CC supervised the project. C-SH, H-CS, H-YL, S-TC, L-PJ, C-TC, T-WH and Y-CC collected plant sample in the field. H-CS, M-HC, Y-ZK, Y-SS and Y-CC mined the SSR primers. C-SH, Y-ZK, Y-SS and Y-CC analyzed the data. Y-CC wrote the manuscript. All authors read and approved the final manuscript.
